# The Hospital. Nursing Section

**Published:** 1906-12-15

**Authors:** 


### The Hospital.

### Nursing Section.

Contributions for this Section of "The Hospital" should be addressed to the Editor, "The Hospital" Nursing Section, 28 & 29 Southampton Street, Strand, London, W.C.

No. 1,056.—Vol. XLI. SATURDAY, DECEMBER 15, 1906.

### Notes on News from the Nursing World.

### OUR CLOTHING DISTRIBUTION.

In order to give the utmost time to those of our readers who have little leisure, and who may be able to send an article at the last moment, we shall receive contributions to our Clothing Distribution up to Monday next, the 17th instant. The whole of the articles, forwarded to enable us to assist the matrons of hospitals and infirmaries in rendering Christmas as happy a period as possible to patients in need of warm clothing, will be on view at the offices of The Hospital on Tuesday between 3 and 5 p.m., when nurses and other friends will be welcome. Since last week parcels are to hand from:—The Misses Clowes, Upper Tooting, S.W.; Nurse Cecil and friends; a large parcel with neither name nor address; Pension Fund Policy 4,091; Clevedon; Miss Ruel; and Nurse N. The Proprietors of The Hospital have contributed a large number of garments. All contributions should be addressed to the Editor, 28 and 29 Southampton Street, Strand, London, W.C., and should have "Clothing Distribution" written outside them.

### A COMPLIMENT TO A COLONIAL TRAINING SCHOOL.

The authorities of Edinburgh Royal Infirmary have paid a compliment to a well-known Colonial training school. Their choice of a new assistant superintendent of nurses has fallen upon Miss Jane Bell, who was trained at the Royal Prince Alfred Hospital, Sydney, New South Wales, and was afterwards sister, night superintendent, and housekeeper in the same institution. She has also been matron of Bundaberg Hospital, Queensland, and matron and superintendent of nurses at Brisbane Hospital, Queensland. The last post she held until July 31 this year. We have no doubt that her appointment will give pleasure to nurses in the Colonies where she has worked, as also to the heads of the school in which she was trained.

### THE HOMES FOR LADY MINTO'S NURSES.

We understand that the arrangements for homes in the several Provinces concerned in the scheme of the Indian Nursing Association are well developed. In the Punjab the existing Home at Kasauli will be utilised, and that at Naini Tal and Bareilly for the United Provinces. The General Provinces have built a Home at Pachmair, where the nurses will be located during the hot weather in turn, and the establishment for the plains will be at Nagpur. Baluchistan, Eastern Bengal and Assam, Burma and Central India hope to have suitable accommodation ready in less than six months. Any nurses engaged in India will be selected by the chief lady superintendent on the spot.

### SELECTED CANDIDATES.

Are the Guardians of a union justified in incurring the expense of having three candidates before them in order to make a selection for one appointment? This question was raised last week at a meeting of a Board in the eastern counties, and the House Committee were taken to task by several of the Guardians because they had pursued this course. It was practically contended that to have a candidate from Epsom, a candidate from London, and a candidate from Birkenhead before the committee was a waste of money. We do not share that opinion. In this instance there were 18 applicants for the post, and we do not think that it was an act of extravagance to ask three to present themselves before the committee, especially as the chairman mentioned that on the last occasion they invited only one, and she was considered to be unsuitable.

### THE METHOD OF CHOICE.

Another question in connection with the mode of election of a nurse in a workhouse infirmary was raised at a meeting of the Rollesby Guardians. In this case; there were three candidates, one of whom, after being interviewed, candidly said that "she did not think much of the place," and the choice of the Guardians was therefore limited to two, although 21 applications had been received. Each of these was the subject of more or less laudatory remarks by individual Guardians, and a good deal of heat was imported into the discussion. Eventually both were formally proposed and seconded, but the chairman urged that this was unnecessary, and that the candidate who obtained the larger number of votes would necessarily be elected. We agree with him, because the method of proposing and seconding candidates is sure to afford excuse for the expansion of the personal element.

### THE DANGER OF COUNTY COUNCIL GRANTS.

At the annual meeting of the Herefordshire County Nursing Association this month, the Bishop of Hereford drew attention to a matter of general interest. The Association has this year received, for the second time, a grant of £150 from the Education Committee of the County Council, and the Bishop, noticing a slight falling-off in the subscriptions, said that there was a risk of the idea spreading that the Association could rely largely upon the County Council for support, whereas he felt that in order to ensure prosperity it must depend mainly upon voluntary contributions. The warning is not unneeded in other directions. It is most desirable, in the interests of the community as well as of nursing, that the County Councils should make grants for the training of nurses in their own counties, but grants of this kind should not be regarded as a portion of ordinary income, and they should stimulate, rather than diminish, the efforts to obtain the maximum of subscriptions.

### A NEW WAY OF GETTING A PIANO.

The superintendent of Newport Union Infirmary has presented to the Guardians a piano, which she has been able to purchase by means of contributions from well-to-do ratepayers in the neighbourhood. This is an unusual mode of providing the nurses with an instrument of music. The superintendent is to be congratulated upon the result of her enterprise, and the nurses upon the fact that they have secured the use of a piano. But it is a growing custom, where a considerable number of nurses are employed, for the authorities to supply them with a piano, and we think that it is preferable to the device successfully adopted at Newport.

### THE IMPORTANCE OF A MASTER KEY.

At an inquest on a spinster of 30, who committed suicide, when she was clearly insane, in a nursing home at Putney, the coroner said that if such homes are to be authorised they ought to be run with a knowledge of the treatment required. This is true, but it was stated that mental cases are not taken at the home, and that the patient only developed insanity after she had been there for some time. The medical man who recommended her removal to the institution is the responsible person in the case. If the matron had been trained at a mental hospital, she would not have told one of the nurses to lock herself in the room with a patient and put the key in her pocket when there was not a duplicate key in the home. In every well-ordered nursing home, whatever the class of patients, a master key to all the rooms should be in the possession of the matron.

### A DISQUALIFICATION FOR THE NURSING PROFESSION.

The Strood Guardians received on Thursday last a letter from Miss M. F. Blower, who had been appointed nurse at the infirmary, refusing to enter upon her duties because she was informed that she would not be allowed to bring her dog with her. The Guardians not unreasonably proposed to claim a month's salary from her in lieu of notice, but on consideration decided not to press the matter. A young woman who cannot be parted from her dog is altogether out of her element in the nursing profession.

### LECTURES AND NURSING ASSOCIATIONS.

At this season of the year it is interesting to note the different kinds of entertainment which are arranged by friends of nursing organisations in order to bring much-needed grist to the mill. At Warrington a concert has been held in the Parr Hall, which was crowded on the occasion; at Darwen there has been a ball, which has now become an annual function, and may practically be regarded as a source of income, the amount varying a little according to the weather; and at Berwick, Sir Hubert Jerningham delivered a lecture, illustrated by lantern slides, in which he related the story of some of his experiences in Japan. The last is a form of assistance which might be more frequently turned to account. As the chairman at Berwick said, nursing institutions are always wanting money, and we believe that the lecture has not yet been utilised as it might be, of course assuming that the subject and the lecturer are sufficiently attractive to secure a fairly good audience.

### A CONTINGENT TAX.

At the annual meeting of the Association of Poor-law Unions of England and Wales a recommendation from the Council reporting on the Registration of Nurses' Bill was considered. It was to the effect that if the suggestion to publish an annual edition of the Register of Nurses were carried out, the nurses ought to pay a small annual registration fee. The Rev. F. H. Higley, of Stepney, took exception to the recommendation on the ground that, as the salaries of nurses were not great, it would be unreasonable to demand the payment of an annual registration fee from them. But he did not succeed in carrying a hostile amendment. We think that in any case the record kept in each poor-law infirmary should suffice to enable a nurse's career to be traced.

### THE NURSES' LODGE.

As we announced a fortnight ago, Miss Hulme has now become responsible for the conduct of a new hostel at 9, 10, and 11 Colosseum Terrace, Regent's Park, N.W. The title is The Nurses' Lodge, and a very moderate scale of charges, by the day and by the week, has been drawn up. Extras and extra meals are also served on conditions to which no exception can be taken. Late leave till midnight is free on Wednesdays, but we do not quite understand why there is a charge of sixpence on other evenings. Is it for the sake of the servants? The telephone can be used for twopence. As to the rules, they are sufficient without being irritating; and the conditions for non-resident nurses are extremely reasonable. Miss Hulme herself is, of course, the superintendent of The Nurses' Lodge. She has already received numerous applications from would-be tenants.

### IPSWICH NURSES' HOME.

At the annual meeting of the subscribers and friends of the Ipswich Nurses' Home it was reported that a scheme for joining the Royal National Pension Fund is under the consideration of the manager. The Chairman, while able to dwell on the success of the Nursing Home which opened last January, and the demand for private nurses, drew special attention to the fact that district and cottage nursing was carried on by the Association last year at a loss of £107. This should not be the case in such a prosperous town as Ipswich, but the difficulty which is admittedly experienced in obtaining sufficient subscriptions, may be partly due to the mistaken impression of people in the locality that the fees received from private patients provide the

Committee with means to defray the salaries of the six fully trained district and the ten cottage nurses.

### SOUTHWELL NURSING SOCIETY AND THE JUBILEE INSTITUTE.

A special meeting of the subscribers of the Southwell Nursing Society was held at the National School on Saturday last, the Archdeacon of Nottingham in the chair. The following resolution was proposed by Dr. Handford, Consulting Physician to Nottingham General Hospital and Medical Officer to Notts County Council, seconded by Mr. Henry Merryweather, Chairman of Hy. Merryweather and Sons, Ltd., and carried *nem. con.*:—"That this meeting regrets that the Committee of the Queen Victoria Jubilee Institute for Nurses, in making charges against the Southwell District Nursing Society, which they have failed to substantiate, in attributing unworthy motives to the Society and its officers, and in declining to reveal their sources of information, have forfeited public confidence, and have left the Southwell District Nursing Society no alternative but to cease to be affiliated to the Queen Victoria Jubilee Institute, and requests the chairman of this meeting to forward this resolution to the chairman of the Council of the Queen Victoria Jubilee Institute." A vote of confidence in the Executive Committee was subsequently carried *nem. con.*

### CONCERT AT GUY'S HOSPITAL.

On Tuesday evening the Guy's Hospital Musical Society gave their first performance of the season in the Court Room. Judging by the prolonged applause, the audience enjoyed and appreciated the efforts of the well trained choir. One of the most interesting features of the evening was the *début*, at the commencement of the second half of the programme of the Orchestral Society. The selection from Mozart, conducted by Dr. H. L. Eason, was well played, and received a deserved encore. During the evening the accompanist, Nurse Margaret Taylor, was presented with a charming bouquet from the members of the societies as a token of their appreciation. Refreshments were served in the adjoining room for the performers, after which the conductor, Mr. Henry Taylor, and Nurse Taylor were thanked in a most cordial manner for their excellent services.

### NURSES' NEEDLEWORK GUILD.

The annual meeting and show of needlework of the Nurses' Needlework Guild was held this year at the Howard de Walden Nurses' Club on Friday last. Many nurses and other friends of the Guild were present.

### NEW MATRON OF AN INDUSTRIAL COLONY.

At a meeting of the Metropolitan Asylums Board on Saturday Miss Jeanette Ferrier was appointed, out of 30 applicants, matron of the Industrial Colony at Darenth Asylum. Miss Ferrier was trained at Bolton Infirmary. She has held the posts of sister of enteric wards at Ruchill Hospital, Glasgow, for two years; night superintendent at the Royal Victoria Hospital, Belfast, for one year; assistant matron of Inverness District Asylum for two years; and assistant matron of the hospital at the new Middlesex County Asylum at Napsbury, St. Albans. Miss Ferrier's long and varied experience should admirably qualify her for her new position.

### PRESENTATION OF BADGES.

At Bradford the other day there was an interesting ceremony in the out-patients' hall of the Royal Infirmary, the occasion being the presentation of gold and silver badges to the probationers who had attained the highest number of marks in the recent examinations. The function was performed by the Mayoress of Bradford, who was accompanied by the Mayor, and among others present were the matron and assistant matron of the Royal Infirmary, the matron of the Woodlands Convalescent Home, the matron of the Children's Hospital, the matron of the Eye and Ear Hospital, the matron of the District Nursing Institution, and the matron of the Bierley Hill Hospital. The attendance, in fact, showed the wide-spread interest taken in the event. The Mayor, having expressed the pleasure it afforded him to take part in the proceedings, the Mayoress, in an admirable little speech, insisted that whatever life had in store for nurses who were trained, their training must be of advantage to them and to others. She subsequently handed gold medals to Miss Dennison and Miss Jameson, and silver medals to Miss Howard and Miss Redfern.

### THE CABMAN AND THE DISTRICT NURSE.

At the annual meeting of the Brighton, Hove, and Preston District Branch of the Queen's Institute, the Rev. A. R. C. Cocks told the story of a cabman who, to his knowledge, had refused to accept payment for driving one of the nurse's patients to the hospital because another nurse had carefully attended to his wife when she was ill. This is a practical proof of gratitude which we quote with much pleasure, though we have reason to believe that it is not an isolated instance, and that cabmen have frequently adopted this useful and self-denying mode of acknowledging services rendered by nurses.

### EMERGENCY NURSES FOR SUSSEX.

The annual meeting of the Sussex County Nursing Association was held on Wednesday last week at Lord Brassey's house in Park Lane, Lord Edmund Talbot, M.P., presided, and several other members of Parliament were present. The honorary treasurer, Mr. A. A. Wace, in seconding the adoption of the report, which shows that 33 local associations, covering 82 parishes, and employing 50 nurses—of whom 11 are Queen's Nurses—are now affiliated to the organisation, expressed the hope that it might shortly be able to extend its usefulness by supplying emergency nurses. He appealed for gifts for a library, which was started in a modest way, and which is being much appreciated.

### SHORT ITEMS.

Last week at Stokesley, in Yorkshire, Miss Mary Amelia Normington, a trained nurse, aged thirty-three, who returned home from America about eighteen months ago, poisoned herself by misadventure with carbolic acid and died in great agony.

### The Nursing Outlook.

"From magnanimity, all fears above;
From nobler recompense, above applause,
Which owes to man's short outlook all its charm."

### THE CURSE OF PRESENTS.

Our forbears and sires held it to be an insult to be offered a present by any but intimate friends. To-day, judging by the experiences described by several correspondents last week, in some institutions present-giving has degenerated into an organised system of extortion, or has become a dangerous absurdity. Good fellowship and liberality, towards one's friends and intimate colleagues, at Christmas time, are one thing; but a system for collecting money from employees for gifts to their employers and superior officers, including an intimation of the amount expected from each, is quite another. The first may be admirable and even helpful. The second may become a tyranny, and should be regarded as a degradation by each recipient of any such gifts. We are glad to notice, that, only one of the matrons, who has written on the subject, is in favour of systematic present-giving in institutions. But, as the exception, "Sister Ellen," only favours gifts to the matron, she seems to us to put herself out of court. The practically unanimous view, then, appears to be, that, it is each matron's part to tell her nurses, as a body, that she does not consider the custom a good one, and that it is her wish that official presents should cease to be given. We hope that every matron may have the courage to take this course, for then Christmas-tide throughout the institutions of the country, is calculated to prove, in fact, a season of peace and goodwill. Under the existing system of present-giving, in some institutions, it must be exactly the reverse.

Official present-giving, after all, especially where it has become organised by custom, as a system, is a matter of business. Great business establishments, clubs, and other communities of men have had to recognise this fact, and it is a common practice, now, for the directors or committees to take charge of the lists, on which every Christmas tip or contribution of the kind is entered, and then to apportion the total amongst the employees, on a plan they consider to be equitable. So it is clearly the duty of every committee of a hospital or institution to take counsel with their senior officials, and to make definite regulations on the subject of presents or gifts at Christmas and other times. The extent of the evil, in some institutions, has been made clear by our correspondents. Fancy the misery of the young nurse in training in an institution, where she finds herself "almost obliged to contribute to presents," to "the matron, assistant matron, night sister, home sister, ward sister, home maids, dining-room maids, porters and postmen." It seems improbable, that, the junior members of the staff, who contribute to lists passed round in favour of all these people, could, in the whole, contribute less than two months' salary. The consequence is, no doubt, as our correspondents state, that many who are near and dear to the givers have to be put off with little or nothing, whilst the givers may be forced into debt. But this is not the whole of it, for sisters and nurses and other residents have, sometimes, to spend money freely on the decoration of their wards, and the provision of other things in connection with the Christmas festivities. The bare statement of these facts should convince every intelligent committee of management, that, present-giving may degenerate into a curse, for it may not only act unfairly upon the weakest and poorest, but it may affect harmfully the character of many members of the staff.

As a matter of business, therefore, and in all equity, every committee ought to promptly look into this question. It is their plain duty to provide, that, the institution under their management is freed from the evils complained of, by the enforcement of a few common-sense rules, such as those suggested by "X.Y.Z." Is there any committeeman, or matron, or superintendent, who, on calm reflection, will countenance collections—collections which "seem to be perpetual, for gifts to doctors and sisters on leaving, annual presents to the matron on her birthday and at Christmas," and so forth? Again, let every committee consider what Christmas decorations may mean, as represented by expenditure in time, money and labour, to the sister of a ward and her staff, especially when the sister, in addition, gives teas two or three times in the Christmas week, whilst she may be paid at the rate of from £30 to £40 a year. All these things are wrong as a matter of business, and, what is more regrettable still, they would become wholly unnecessary, if the committee and secretary always took an active interest in the welfare of the inmates. In the latter case it is the custom for the committee and their friends, with members of the visiting staff, to raise a fund to defray the whole cost of the Christmas festivities and decorations. This is only fair, for Christmas proves an asset for many a hospital. The festivities and decorations attract public notice and win the sympathy of outsiders, who may, in this way, become liberal contributors to its funds. This seems to us a case where harm has been done, in the past, from want of thought, rather than want of heart. We hope, that the chairman of every hospital, and all the more active members of each hospital committee, will make it their business, to take up these questions, everywhere, throughout the country. If they do their duty, promptly, a remedy for the existing abuses will be found, and a few simple but necessary regulations will soon appear in the statutes.

### Nursing in Tropical Climates.

By Andrew Duncan, M.D., B.S., M.R.C.P., F.R.C.S., Fellow King's College, Lecturer on Tropical Medicine at the London School of Tropical Medicine, and the Westminster Hospital.

### II.—ON THE EFFECTS OF THE SUN, AND THEIR PREVENTION.

In treating of the various tropical diseases and the duties of the nurse as regards them, I propose with each disease to narrate shortly their clinical symptoms, then to discuss the facts of their causation which it is desirable a nurse should know, as without such knowledge she cannot possibly understand how to prevent them, and finally to discuss the preventive and nursing treatment of each.

### 1. Heat-stroke.

The first enemy to health that an immigrant into the Tropics encounters is heat, and therefore we will begin our subject with the effects of heat. Now the heat of the tropical sun will be always more or less present; we must know something, therefore, of what it will cause and how to prevent its bad effects; for, should an intending worker in tropical climates be incapacitated at the outset of his or her career, the ambition of those entering on a tropical life may be at the beginning frustrated, for once you are attacked by the sun, ever afterwards you will be more liable to further attacks.

*Clinical Varieties of Heat-stroke.*

The sun's rays may affect an individual in more than one way. The subject will best be considered under the following heads: *A.* Heat Collapse; *B.* Heat-stroke; (*a*) Direct heat-stroke or sunstroke proper; (*b*) Indirect heat-stroke.

*A. Heat Collapse.*—Here the patient suddenly turns giddy and falls. His skin is moist and cool, his breathing hurried, but never stertorous, the pulse small and soft, the pupils dilated, and the temperature at or below the normal; there is also no complete loss of consciousness.

*B. Heat-stroke*: (*a*) *Direct Heat-stroke or Sunstroke.*—There are several varieties of this form. (1) In one the sufferers are chiefly unaccustomed to the fatigue of marching, and they are particularly liable to be seized by it when the skin does not perspire well, as when the air is moist; after the onset of violent headache, the patient continuing his march, falls down in convulsions. Insensibility is absolute, and often incontinence of urine occurs, he has difficult respiration, and his teeth are firmly clenched. (2) In another the subject streams with sweat, becomes paler and paler, with swollen veins, injected eye, quick and shallow respiration, and at length falls to the ground. Consciousness is not generally entirely lost, and if the patient be laid down, and his respiration be relieved of all impediment, revival occurs. (3) The patient becomes extremely thirsty and suddenly falls forward comatose. (4) The patient, after a hot march, is seized with a raking, splitting headache, hourly becoming more and more intense until the greatest agony is experienced. With this there is intolerance of light, and unconsciousness sets in. Should this be recovered from, the intense pain in the head will last without cessation for from six to eight weeks, the only remission being at sundown.

(*b*) *Indirect Heat-stroke.*—Here the patient is not attacked in the open, but in his hot, close bungalow. He becomes pale, there may be nausea, colic, and incontinence of urine. Next follow convulsion, with cyanosis dyspnœa and insensibility. The breathing is stertorous, the pupils contracted, and the temperature of the body may reach 108° to 110° F., and remains high for some time after death.

Such are very briefly the clinical signs of the effects of the tropical heat on patients who are affected by it.

*Etiology.*

Several theories have been put forward to explain the occurrence of heat-strokes, such as: (1) The caloric theory, which attributes the attack entirely to the action of intense heat. (2) The poisoned-blood theory, according to which the heat causes the blood to become poisonous to the nerve cells. (3) The microbic theory, or that theory stating the symptoms are due to the action of a microbe. (4) The theory that the actinic rays of the sun cause the attack. This last theory, in my opinion, is the correct one, as I shall proceed to show.

*Preventive Measures.*

1. *Alcoholic drinks ought never to be taken when exposed to a hot sun.* Tea and coffee are the drinks *par excellence.* The sun's action diminishes the action of the skin, lessens nervous activity, causes less carbonic dioxide to be exhaled, and induces cardiac paralysis. Now the action of tea and coffee is exactly the reverse to all the above, they also counteract the onset of fatigue, so deadly a factor in heat-stroke, and hence are clearly indicated when much muscular exertion is undertaken. Hence, if a nurse has to make a journey in the heat of the day, let her fill her water-bottle with cold tea.

2. *Dress.*—As to the head-covering, the nurse should first of all remember never to go out in the sun with her head unprotected. As regards the best form of headwear, she should possess for the day one of the numerous forms of solah-pith topee—the uglier they are in shape the more efficacious they commonly are—but with a puggaree around them they may be made presentable. They can be obtained in India; for the voyage out a double Terai hat should be purchased, as this is a very comely article to wear in the evening in the Tropics. An excellent form of topee to get is that known as the " Cawnpore tent club helmet," purchasable at Cawnpore and other places. She should also wear neutral-tinted spectacles, these afford the greatest relief to the eyes from the glare. She should have a woollen pad sewn into the dress over the spinal column. The dress itself should be loose round the neck and chest. The best material is of light wool, the latter being but a slow conductor of heat and does not get saturated with perspiration like cotton.

Also, if you place two thermometers exposed to the sun and cover up one with a cotton sheet and the other with a woollen blanket, you will find the former to register a higher temperature than the latter. The advantage of not having one's dress to fit tightly is well shown by an instance quoted by Surgeon-General Sir A. De Renzy. At the capture of Rangoon in 1852 " of two sets of men who were alike in all other respects, one fought in shirt sleeves under an intensely hot sun, the other in blue cloth tightly fitting coats; many of the latter began to drop down insensible shortly after coming into action, their commanding officer being amongst the number."

I have said previously that the theory of heat-stroke that seems to me to be the correct one is the chemical one. To explain this it is necessary to describe the effect of the passage of the sun's rays through a prism. After passing through a prism the sun's rays are split up into what is termed a spectrum, which consists of a band of the following colours in order—namely violet, indigo, blue, green, yellow, orange, red. This is the visible part of the spectrum, for beyond it at either end we have evidence of rays which are invisible, namely beyond the violent end there are rays known as chemical or actinic rays, for the effect they have in decomposing certain salts, as nitrate of silver as in photography. The chemical action of light chiefly depends on these so-called actinic rays. Beyond the other end of the spectrum are what are known as the heat rays. Now any fabric dyed red or orange will filter off the actinic or chemical rays.

An officer well known to me had suffered from sun-stroke in the Afghan war and had three subsequent attacks in India. As each hot weather came round he was affected with severe headaches on and off, until one day he had the good fortune to read a letter from Major F. N. Maude, R.E., in the daily paper. This officer had had repeated attacks of sun-stroke, and he formed the idea that the dangerous rays of the sun in this respect were the actinic rays, which pierce practically through anything except always a layer of colour interposed as a filter. Dark red or yellow is such a colour, as every photographer knows. He therefore used a red or yellow lining to his hat and coat, precisely for the reason that a photographer develops his plates under red or yellow light. The result was that he no longer felt the sun, and remained free from its ill-effects, except on one occasion when a brother officer removed this lining unknown to him from his helmet, when he was seized with severe headache and the initial symptoms of sunstroke on going out to his work shortly after. My friend followed the same line of treatment and never afterwards felt the sun. He told several people of this and they also had the same fortunate experience. Hence the nurse should line her helmet with red flannel, and also cover the pad down the inside of her dress over the spine with red. So much, then, for the question of prophylaxis. As regards the actual treatment of a case, the patient should be moved into the shade and his clothes opened about the neck. Cold water from a " mussack " should be poured on to his head and neck. Ammonia should be applied to his nostrils. The douche is to be repeated until a favourable effect be produced. A turpentine enema should be given and a large mustard poultice applied to the chest. The late Dr. Norman Chevers strongly advocated "planing" the head with ice. This, however, should not be done when the skin is cold and the pulse feeble. The attending physician will give his directions to the nurse as to the carrying-out of these measures.

This lecture, it will be seen, has been chiefly occupied with the measures the nurse herself should take as regards the prevention of heat-stroke. But these of themselves are of chief importance, in order that her services may be retained intact for the benefit of her patients from other tropical diseases.

### The Nurses' Clinic.

### CUT THROAT AND FRACTURES OF SPINE.

*Fracture of the lower jaw*, the symptoms of which are loosening and irregularity of teeth, crepitus, dribbling of blood-stained saliva, and impairment of speech, and great pain in attempting to open the mouth.

The patient will be kept entirely on fluids, probably administered by nutrient enemata for a few days, and then sucked in through the teeth, to prevent all mastication or movement of the jaw. The fluid diet will probably be continued for two or three weeks. After four weeks a little movement of the jaw is allowed, and after five weeks the union ought to be complete. These cases are very trying to nurse, as it is most difficult to keep the patient from mastication. The patient also is in great pain and discomfort, and consequently inclined to be irritable and hard to please. The bowels should be opened by enema, and the mouth washed out with antiseptic lotion, which should be allowed to run in gently on one side and out on the other.

*Cut Throat.*—As most nurses come across these cases during their training, it will not be necessary to say much about them. The first thing to attend to will be the arrest of hæmorrhage. The nurse should prepare fine sutures, and a tracheotomy set for the surgeon, who will require it should the trachea be severed. The patient should be raised on pillows, with the head bent forward, and secured in this position by bandages. He should be placed in a warm room, the air being moistened by a steam-kettle. The diet will be fluid. Often the patient refuses to swallow, in which case he can best be fed by a small tube passed through the nose into the stomach. All these cases must be closely watched as they frequently attempt their life again, especially in the case of suicides. When the air passages are severed a frequent complication is broncho-pneumonia. This may be caused by the cold air entering direct into the lungs, or by inhaling septic matter from the wound, or by particles of food entering the air passages in consequence of the loss of feeling in the glottis. Broncho-pneumonia is a most serious complication and frequently ends fatally.

*Wounds of the Spine.*—Penetrating wounds of the spine are of very rare occurrence in civil work. They may be caused by gunshot wounds or stabs with sharp-pointed

THE NURSES' CLINIC.—*Continued.*

instruments. These latter ones will mostly be in the neck, as it is easy to penetrate to the vertebræ. In most cases there are no immediate symptoms, the signs of hæmorrhage between the bone and the dura mater may develop later on. In cases where the membranes have been penetrated, the cerebro-spinal fluid will escape from the wound. Should the spinal cord be completely divided paralysis of sensation and motion will occur in parts, according to where the division takes place. Complete asepsis of the wound must be principally aimed at. Place the patient flat on the bed, with no pillows, and preferably face downwards. If there is much cerebro-spinal fluid escaping, a gauze bag filled with boric powder put over the wound will help to keep the parts clean.

*Fracture of the Spine.*—In this injury no attempt will be made to reduce the fracture, but the patient must be kept absolutely still. Very frequently the patient will be put into a spinal jacket.

Fractured spine case when brought in should be laid on the bed, the clothes cut, and removed very gently. Then he should be wrapped in a blanket, and hot-water bottles applied, great care being taken to prevent them burning the patient, and hot beef-tea, coffee, or brandy given. The head must be kept low. The patient should be placed on a water-bed provided with a mackintosh and draw-sheet. The two dangers to guard against in these cases are bed-sores and cystitis. Bed-sores will form in any places, not only when there is pressure, and it needs the mast attentive and skilful nursing to prevent them, but even with the greatest care they will come sometimes, and of course should be reported immediately to the doctor. Scrupulous cleanliness and attention to the draw-sheet to see that it is not wet or soiled, and that there are no wrinkles or crumbs of bread in it. If in a jacket, the patient may be very gently rolled slightly from one side to the other, the back being well supported by pillows. The bladder should be emptied every six or eight hours with a soft rubber catheter, to guard as much as possible against cystitis setting in. The catheter should be carefully washed and disinfected before and after use, to prevent septic germs being introduced into the bladder, and should be well oiled before introducing. After the catheter is removed, the patient should be carefully washed and dried. The bowels would be opened by enema or castor oil. If diarrhœa appears, it is a great tax upon the nurse's skill to keep the patient dry and clean. Starch and opium enemata are often ordered for this condition. The diet should be nourishing, but easily digestible. It is best to have a flannel bed-gown, open down the back, that can be just laid over the patient, and not under the back at all.

It will be necessary to have three people to help with washing the patient's back. Two to roll the patient on to the side and support the back well, while the third one does the washing. If there is incontinence of urine, arrangements can be made with the water-bottle in the case of a male; with a female patient it is much more difficult, but much may be done with absorbent wool, tow, and jaconet. These cases are most trying to the strength and patience of the nurses, as they are generally very long cases and need continual care and attention and need much cheering up and amusing.

### Incidents in a Nurse's Life.

### IN A BANGALORE MILITARY HOSPITAL.

An early morning, more like one of England's best May days, warming up to a July's warmth—so Christmas Day came to us in our hospital. We had a busy day, yet nothing to be compared to the work entailed in a hospital at home; and there was no Covent Garden to visit. Decorations were chiefly of plants brought up from the garden by the mahli, and placed on window-sills, along the verandah, and by the entrance to the sister's duty room, where the medical officers, their wives, and the padre were to be regaled with tea after the patients' tea was over. A few annas to the bungalow mahli brought in roses, hibiscus, asters, chrysanthemums, and flowering creepers, and the decorations were soon done by the sister on duty and the patients, who, in their zeal to help, needed a restraining hand in the mixing of colours, the effects produced being sometimes somewhat appalling.

After the dinner at 1.30—ordinary diets and milk-puddings, as on other days—the sisters went with the medical officer to visit the nursing orderlies at their meal, and drink to their health in port, soda-water, or lemonade, served to each by the corporal. Their bungalow was beautifully decorated with strings of coloured paper, starting from a huge rosette in the centre of the ceiling, which rosette, I was told, had a helmet as foundation. Two brown Army blankets hung up had respectively "Long Live the King!" "Long Live the Queen!" done in cotton-wool stuck on with gum; and a green shield had in gold lettering, "Success to the Nursing Sisters and Medical Officer." The dinner had been entirely prepared by the orderlies, and they had done "night duty and day duty" on the boiling of the puddings, having taken turns in seeing that they kept boiling for twenty-four hours about a week before Christmas Day! One pudding was sent to the sisters' wards to be distributed amongst the patients. Fortunately there were no very bad cases, and the kindly thought was much appreciated. Their bill of fare was a plentiful one—a huge turkey, fowls, ducks, fruit, and, on a side-table, a huge joint of beef, carved by one of the hospital cooks.

Then we started in earnest for the great event of the day—a tea for about fifty, as, besides our own patients, the hospital police, orderlies, and patients from the lower wards were invited.

Our butler and chokra came up with the bullock-bandy laden with good things, and were warned to keep a strict eye on everything: the ward boys are such terrible thieves. The patients also were put in charge of the tables. Many sat and admired, whilst others took the laden plates and dishes from the sisters and arranged them on the tables with the greatest pride. One sister had been seized with the happy idea that ham would be a great treat (it is rather a luxury in India), and by dint of careful management the little pile of rupees was made to include two big hams. We paid our own cook to boil them the day before, and one sister kept a careful eye on them to see that they did not stop boiling. People at home would wonder at the marvels in cooking done over funny little brick furnaces fed by wood or charcoal; often an ordinary large flower-pot is used. We were very proud of our tables when finished—piles of bread-and-butter, some spread with potted meat, jam, huge cakes, pyramids of oranges, loaf sugar (a great treat to the Tommies), and cups and saucers instead of the usual mugs.

I carved the biggest ham, and raced an officer, who tackled the other. The plates coming again and again testified as to how it was appreciated, one bone being picked clean and the other having very little covering.

All the medical officers turned up and saw that plates and cups were kept filled. "It's the best Christmas as I've seen in India," was the general verdict when, the inner man satisfied, tongues were loosened as a generous supply of smokes was passed round.

After a short speech from the padre we left the men enjoying a gramophone, and retired to the duty-room to enjoy our own tea.

### Femoral Hernia in Private or District Work.

EXAMINATION QUESTIONS FOR NURSES.

The question was as follows : " In the case of inguinal or femoral hernia (in private or district work) what steps should you take to alleviate suffering pending the arrival of the doctor? Differentiate in your answer between the necessary treatment that should be tried for the three kinds of hernia—reducible, irreducible, and strangulated—and remember to keep strictly to your own work, and not to usurp that of the doctor."

First Prize.

Place patient in bed, keep knees drawn up, so as to relax abdominal muscles, by placing pillow or cushions under knees. If hernia is reducible it may go back of itself, or with very gentle pressure; if not, apply flannels wrung out of hot water, or ice, broken in small pieces and tied up in a piece of jaconet, or any thin waterproof material, over hernia, or place patient in a hot bath in a recumbent position, with knees drawn up. Keep up the temperature of the bath, and see he does not get cold after leaving it. When hernia is once back in place, place a pad of lint, or two or three folded handkerchiefs, over opening in abdominal wall, and keep securely in position with a spica bandage. If patient has a cough or is vomiting, always support the hernia with gentle pressure of the hand. If the hernia is irreducible continue to keep patient in recumbent position as above, and apply flannels wrung out of hot water. If strangulated, shave and cleanse the part, apply fomentations, and prepare for immediate operation. Keep him warm with hot-water bottles or hot bricks, wrapped up well in flannel, and keep very quiet. As there is more or less vomiting in all three stages, do not give any food until the doctor gives permission, but keep the mouth moist by wetting the lips with water. In strangulation fæces is often vomited, therefore it is necessary to cleanse the mouth with some mild antiseptic, such as boracic or a weak solution of Condy's fluid.—" Phœbe."

Second Prize.

In each case put patient to bed, with knee pillow to relieve abdominal muscles; raise the foot of the bed on blocks or bricks. Treat patient with great care, because if hernia is not already strangulated it may become so with careless treatment.

1. *Reducible Hernia.*—Make patient warm and comfortable, apply gentle pressure over hernia when patient coughs or moves. If hernia slips back put on a firm pad and bandage, or in case of a baby a truss made of a skein of white wool if one at hand.

2. *Irreducible Hernia.*—Make patient warm and comfortable. Take T.P.R. If much pain apply ice (if procurable) in ice or sponge bag, or flannel wrung out of hot water. Keep mouth moist, but give no food before doctor's arrival in case operation is necessary.

3. *Strangulated Hernia.*—As patient will probably be collapsed, apply quickly hot blankets, hot bottles of any sort, or bricks, etc. Take T.P.R. Apply crushed ice in bag with care, or fomentations (this will help to reduce hernia). Save vomit for doctor's inspection. Both in this and irreducible hernia find out when bowels were last opened, and if flatus is passed. Moisten mouth, but give no food. If patient likes, keep pressure of clothes from abdomen with part of box in place of cradle. Start preparations for operation, as it will probably be immediate.—E. A. Y.

The Quality of the Papers.

This is much improved since the answers to last month's question; but nurses must remember that this improvement is a plain proof that they did not think sufficiently before answering in October, writing their papers hastily, without taking the trouble to consider the importance of the question all round.

The answers of the first and second prize-winners are almost equally good. " Phœbe," who came out second last month, bears off the victor's palm this time because she speaks of the desirability of applying either heat or cold to a reducible hernia as well as to an irreducible one.

Honourable Mention.

This is gained by " Central," " Ellen," " Little Somerset," " Kathleen," and " Daisy," who all send good answers.

Question for December.

What treatment should you apply (from a strictly nursing point of view only) to a severe case of rheumatic fever? N.B.—The answer must not touch upon drugs or any thing to do with the medical man's province, but deal with the subjects of bedmaking, washing, and dieting, if the latter is permitted by the doctor. The Examiner.

Rules.

The competition is open to all. Answers must not exceed 500 words, and must be written on one side of the paper only, without divisions, head lines, or marginal notes. The pseudonym, as well as the proper name and address, must be written on the *same* paper, and *not on a separate sheet.* Papers may be sent in for 15 days only from the day of the publication of the question. All illustrations strictly prohibited. Failure to comply with these rules will disqualify the candidate for competition. Prizes will be awarded for the best two answers. Papers to be sent to " The Editor," with " Examination " written on the left-hand corner of the envelope.

In addition to two prizes honourable mention cards will be awarded to those who have sent in exceptionally good papers.

N.B.—The decision of the Examiner is final, and no correspondence on the subject can be entertained.

Any competitor having gained three prizes within the current year shall be disqualified from taking another until 12 months shall have expired since the first prize was gained.

### Nursing in a Plague Hospital at Zanzibar.

It was quite a new experience to me on leaving one of our up-to-date Scotch hospitals and being transferred to Zanzibar for plague duty to be driven three miles every day to and from the hospital wards in a carriage and pair—the " pair " consisting of two fine mules—with a coachman—" syce "—and footman, the latter standing on a step behind us, and, when occasion required, jumping down and running in front to drive away the tardy natives.

My friend and I were told off for night duty, so that every night at 7.15 p.m. we were driven through the native quarter to the hospital, and back again to our quarters at 7 a.m.

It was impossible to get the carriage up to the hospital itself, so we had to walk about a quarter of a mile along a path with grass on either side, and where snakes were wont to lie—though we never actually encountered one during the walk. It was usually very dark, so that as soon as we got out of our carriage we cried out, " Butti lau "—" Bring a light ! "—and after repeating the cry several times a porter appeared running with a hurricane lantern, and we were thus escorted to the hospital accompanied by another attendant who carried our tray of provisions for the night.

At first everything seemed terribly strange. Never shall I forget the first night. My heart sank at first when I looked round on those poor patients and felt that neither they nor the attendants could understand one word I spoke. Fortunately my friend can speak some Hindustani, and she taught me some useful phrases. We soon seemed to know the patients and the treatment ordered by studying their notes and charts. There was no written report until after we arrived.

The hospital is surrounded by a verandah, on which we used to sit when we had a few minutes to spare and where we boiled our water on an oil lamp for tea. We were not left alone, as mosquitoes, ants, and other pests sought to make our acquaintance, and indeed troubled us incessantly; centipedes and other crawling creatures came up the steps and meandered about until they were summarily dismissed!

The kitchen was separate from the wards, and all water was heated and milk boiled on a wood fire. The cook slept there on a wooden plank, so she was ready when needed.

Our patients, of course, needed a great deal of attention. The plague being of the bubonic kind, the treatment consisted of hot fomentations applied to the buboes in the axillæ and groins. Feeds of milk and Brand's essence of chicken were administered two hourly, and in most cases stimulants were also given. The patients were often delirious, the temperature of the body frequently registering 105° to 109° F. We had a convalescent ward about 300 yards from the hospital; but most of these patients slept all night, and we paid them a visit and took their temperatures in the morning. The hospital was in charge of a European doctor, and he had a qualified native assistant, who slept on the premises.

By 7. A.M. I can honestly say that we were tired and quite glad to be relieved by the day nurse and to jump into our carriage and drive "home" to breakfast, bath, and bed. The sun was fearfully hot even at that hour of the morning, so that we had to wear our blue glasses and sun topees.

There is something very calm and peaceful about night in the tropics, especially in the far-away quaint little island of Zanzibar, where the great palm-trees sway gently to the stars. But there is also a continual humming and buzzing from the insect world, which is always apparently awake. Birds and beasts (except pariah dogs) and flowers are mostly asleep, but mosquitoes, frogs, bats, and grasshoppers are very much awake.

At certain seasons of the year the Southern Cross may be easily seen, and as the night advances it passes higher and higher across the sky until at last it sets. The stars are very bright, and at full moon one does not require artificial light in order to see to read.

There always seems a specially sad element in the nursing of plague. One longs to be able to do something for the poor natives, who are so ignorant, and even hide away their sick rather than send them into hospital. During one month last year out of 135 cases of plague 104 died.

There is also a brighter side, for the patients who come to us do seem to realise that we are only seeking their good, and as a rule are willing to undergo whatever treatment may be ordered.

As a rule native patients are not ungrateful, and they never forget an act of kindness. Even after the death of a patient in hospital the relatives and friends appear to realise that everything possible has been done to try and save life, and by degrees they learn to understand that we wish to be their friends and not, as they formerly thought, to do them harm.

### To Nurses.

We invite contributions from any of our readers, and shall be glad to pay for "Notes on News from the Nursing World," "Incidents in a Nurse's Life," or for articles describing nursing experiences at home or abroad dealing with any nursing question from an original point of view, according to length. The minimum payment is 5s. Contributions on topical subjects are specially welcome. Notices of appointments, letters, entertainments, presentations, and deaths are not paid for, but we are always glad to receive them. All rejected manuscripts are returned in due course, and all payments for manuscripts used are made as early as possible after the beginning of each quarter.

### Everybody's Opinion.

[Correspondence on all subjects is invited, but we cannot in any way be responsible for the opinions expressed by our correspondents. No communication can be entertained if the name and address of the correspondent are not given as a guarantee of good faith, but not necessarily for publication. All correspondents should write on one side of the paper only.]

### "CRUEL AND DEGRADING CHRISTMAS PRESENTS."

"Sister" writes: I should like to reply to the letter in this week's Hospital written by "Charge Nurse." I have been attached to a large provincial infirmary for several years as probationer, staff nurse, private staff nurse, and sister—most probably the institution referred to—as the naming of the subscription lists coincide. Such presents are given, but are quite voluntary. No one is asked, and no one would be thought mean if she had the moral courage or grit to say she could not afford it or would rather not. We all look upon our presents to matron and others as a means of conveying our affection and gratitude for innumerable attentions and kindnesses during the year. We give as friend to friend—*not* because it is expected or customary. I think the giving of presents to those we like is most delightful. Why should it differ because one happens to live in hospital? We should be very sorry if any of our friends thought we gave them presents under compulsion. One might as well say wedding and birthday gifts are scandalous. I quite agree with the letters written by "Sister B" and "One Who Likes Fair Play." Christmas evidently does not give much pleasure to "A Poor Penniless Pro." or "Charge Nurse"; they must be very weak-willed and foolish to give to people when they cannot afford it.

"E. M. M." writes: I should like to give my sympathy to "A Poor Penniless Pro.," for it becomes quite a compulsory tax at Christmastide for subscriptions for the seniors' gifts. As regards the purchase of the gift being a pleasure, I can from experience say that the poor pro. is never consulted at all in the matter. She merely gives her subscriptions, does not know what is bought until Christmas Eve arrives; and she is just called into sister's room as a matter of etiquette to look at the gift. It certainly would appear very mean not to give towards the gift, even if you can ill afford it. I am delighted that this brave pro. has had the courage to make this question public, and I hope that matrons in private institutions, as well as hospitals, will pay heed to her protest. As to a matron providing posts for her nurses and buying tickets for theatres, it is only her duty to do so when her nurses show their loyalty by using up all their strength in the wards to give every satisfaction they can. It seems rather selfish of a sister to begrudge a poor pro. a little pleasure for only saying that Christmas subscriptions were a tax. Perhaps a good many sisters forget that they were once pros. themselves. I trust that matrons will help to teach the nurses to save for rainy days.

"A Metropolitan Asylum Board Hospital Nurse" writes: I was very pleased to read "A Poor Penniless Pro.'s" letter in your columns, as I think it quite time something was done to put an end to this most demoralising practice of present-giving to people in authority. Of course during training the probationer's one aim is the gaining of the much coveted certificate, and she certainly cannot afford to risk anyone's displeasure by refusing to subscribe to the many subscriptions thrust before her. Consequently the usual home gifts are sacrificed also, and what is of far greater importance, the girl's principle suffers, as, although she feels compelled to give for fear of being thought mean, it is entirely antagonistic to her principle to do so. Personally, I think to be asked to subscribe to presents to those we work with, and for whom we admire and respect, is a perfect outrage on the finer feelings of womankind. We all know the feeling of being offered 10s. by a friend of a patient who we have nursed. Yet to give a present to the matron, doctor, and sisters is just the same thing. Then, again, I have repeatedly been asked to subscribe to presents for sisters whom it has been impossible for me to respect. I am sure even the most loyal amongst the nursing profession cannot deny that it is impossible to respect some people whom we are compelled to work under. I might also add that we did not give our matron and doctors presents, but it was customary to give the sisters and night superintendents Christmas presents. I think the remedy for this most demoralising evil rests with the higher powers in giving the nurses to understand that they refuse to take presents. You can do much by bringing it to their notice in your valuable paper.

### MALE NURSES.

C. Bryant, late ward master Royal Navy, writes : I venture to think the statement made in your columns to the effect that the National and Military Hospitals are the only hospitals where men are trained and certificates granted is misleading. There appears to be a body of men almost unknown, except at our naval towns. No mention is made of the nursing department of the Royal Navy—viz., ward masters, sick bay stewards, and attendants. These men are male nurses in every sense of the word. On joining they are sent to one or other of the large naval hospitals—Haslar, Devonport, or Chatham. After passing the required examination in medical and surgical nursing they are employed on board ships of the various fleets and naval hospitals at Gibraltar, Malta, Ascension, Simon's Bay, Hong Kong, and by the large number of ships and hospitals mentioned it is easy to realise the number of male nurses who are required for the sick bay department in the Royal Navy.

[But these train only for the Navy.—Editor of The Hospital.]

### ANOTHER STRANGE DISMISSAL.

A. E. Middleton writes : In your issue of November 17 I noticed, under the heading "A Strange Dismissal," a letter from a district nurse, and it has occurred to me to relate the story of a stranger dismissal than the one "District Nurse" tells about, and which came under my notice lately. A Queen's Nurse had been working away satisfactorily for a few years in her district, when one day she received notice to leave in a month's time—the only reason given being that a "change" was desired. The nurse naturally asked for a reason, but was told that the Committee were acting within their rights in terminating her engagement and that they declined to state their reasons for doing so. Without going into all the details of the case, suffice it to say that nurse's reports by the inspectors had always been most satisfactory, all the doctors spoke well of her, and no patient had ever complained. On the face of it this bears evidence of oppression and grossly unfair treatment, and it would be interesting to know whether any of your readers can suggest a remedy or devise some means whereby such treatment of a capable nurse who attends properly and faithfully to her duties would become impossible.

### PATIENTS' CLOTHING AT HOSPITALS.

"E. W." writes : I wonder if it has ever occurred to the matrons of our large London hospitals, or, rather, of those hospitals where a room is not provided for storing the clothing of the patients who come in for treatment, that friends have not only to take it home, but bring it again when the patient returns home. I know that it is difficult always to arrange matters to please every one, but I think that perhaps something might be done for nurses not living near London. I took a patient a short time ago for an operation, and was rather astonished to find that I had to carry home the large shawl I had her wrapped up in and the bed jacket I had lent her, as it was a pouring wet day, and I had a parcel of my own, my umbrella, and my cloak to carry, also to travel on two companies' railways, and walk three miles from a station to my home. I felt that the rules might not be quite like the law o fthe Medes and Persians, which altereth not. I was delighted to hear from some of the patients how very comfortable they were in that particular hospital, and they spoke warmly of the kindness of the sister and nurses. I felt that it was a pity there should be that one drawback to such a nicely arranged hospital. I thought more about it this week when I saw your appeal for clothing for the in-patients. The bed jacket I lent was a good one and clean, so I am sure she could have worn it, but that, of course, does not matter; it was bringing the clothing home that I considered such a pity.

### IMPROPER FOOD AND INFANTILE MORTALITY.

"A Maternity Nurse" writes : I have been very much interested in reading the article in your columns on "Improper Foods as a Factor in Infantile Mortality." It is a subject I have been intensely interested in since my training at Queen Charlotte's Hospital, where we received splendid teaching in the feeding and care of infants. I think it is so sad the decline in breast-feeding, but I have found that often a prospective mother will have heard that her mother, or some relations of the family, have either not been able to, or did not, breast-feed their babies, and she will think she cannot do it; then I have pointed out the immense advantage to a baby to be breast-fed, and suggested to them a certain diet, avoiding alcohol (I might perhaps say here I have not given a patient while under my care any alcohol, and all the medical men I have nursed the cases for have been satisfied with the results), taking plenty of milk and meat so cooked that the goodness is preserved, and all food that nourishes. Then after delivery I followed up the same course of diet, always, of course, subject to the approval of the medical man attending the case, and as far as it was conformable to the rules of the household, superintended the cooking, so that there was no re-heated meat or warmed up, but freshly cooked, hot meals given to the patient; and in all cases so far I have found the mother has been able to breast-feed her baby up to from three to six months at least—one instance especially, the mother and sisters of my patient were astonished at the baby being breast-fed, for they said she was the only one who had done so from the immediate family. I think perhaps much of the hand-feeding for babies is due to the fact that mothers will not quiet themselves and give the time and attention which I am old-fashioned enough to believe must be given if an infant is to be satisfactorily fed. If only mothers could be brought to realise that when they are feeding their babies their minds must be quiet, the atmosphere of the room calm, and not a lot of talking and laughing, nor yet a gloomy silence, but just attention given to the baby. I have seen some mothers, anxious to get the feeding-time over, trying to hurry up the wee one, which ends in the child not being satisfied, and consequently not being able to sleep, and crying; then they think they cannot satisfy the baby by breast-feeding, and often the failure is only for want of quietness, attention, and the giving up considerable time. Though it may perhaps seem to some irrelevant to the subject, I think the mother-love increases, and mother and child are brought together in a closer bond, when all the claims of maternity are fulfilled. Of course this applies more especially to the well-to-do classes, for I think they are more lax than their less well-to-do sisters, owing to the many social functions they consider they are called upon to attend. I have written this not with any idea to dogmatise, but it is just my experience in this decline of breast-feeding after a few years' nursing, and in which I only feel a beginner; so shall be very glad for any criticisms or further suggestions from those more qualified.

### IRRELEVANT QUESTIONS.

Jenny L. Foster Newton writes from Bingham House, Richmond, Surrey : My attention has been drawn to the paragraph "Irrelevant Questions" which appeared in a recent issue of your paper, and as I was the Guardian who ventured to interrogate the candidates on the subject of total abstinence—I am not aware that any questions were put to the candidates on the other points mentioned in the paragraph, namely, marriage and religion —I feel justified in stating my reasons for considering it my duty to ask the questions which I did. "*Experientia docet*," and I may say that it is not a very long time since I took into my own home an unhappy woman who had recently been dismissed from the position of nurse at a workhouse for drunkenness and kept her under my own supervision until I could collect sufficient funds from persons interested in temperance work to enable her to be sent to a "Home for Inebriates," where she remained for a year. This case is not singular, for I have, during my seventeen years' experience as a Guardian, known other cases among Poor-law officers where, as the result of excessive indulgence in alcohol, voluntary resignation has been the only alternative to instant dismissal. Under these circumstances I emphatically protest against any suggestion that Guardians are asking irrelevant questions when they seek to ascertain the views of any officer, especially of a nurse, in regard to the very prominent and important question of total abstinence. The trend of most employers of labour is distinctly in favour of the worker who abstains from the use of alcohol. In our own homes we desire that our dear ones should be attended by a person whose breath is absolutely free from this alcoholic poison, whose eyelids are not rendered heavy by its use, whose work is not lessened or whose temper is not made uncertain, as is frequently the case with a non-abstainer. Let us give the same to our sick poor as we seek for ourselves. In conclusion, I maintain that a total abstaining nurse, all other things being equal, is to be preferred to one who daily indulges in alcohol.

### BABY'S COMFORTER.

"Nurse G." writes : As a fully-trained nurse and midwife I rejoiced greatly at Dr. Tom F. Bedley's remarks about "comforters." I have been district midwife—as well as parish nurse—here for 2½ years, and find the abuse of the "dummy" terrible. The children have them from birth until after they go to school. I should like to suggest to "Trained Nurse" that, though there is great reason in what she says, the question of example may not have struck her—even if her babies do not have "dummies" in the street, the servants of the homes where they are brought up will be the mothers in the district in a few years' time. It is a shocking fact to me that these abominations are seen in the children's wards of hospitals. We learnt when children that "example is better than precept," but a cane is sometimes more effectual than either. Therefore I hope the day is not far distant when to sell or use a "dummy" will be punishable by law.

### THE CASE OF MISS HULME.

Miss Annie E. Hulme, superintendent of The Nurses' Lodge, 9, 10, 11 Colosseum Terrace, Regent's Park, N.W., writes : May I be permitted through the medium of your well-known paper to express my sincere thanks to the press for having placed my case in so able a manner before the nursing profession? May I also take this opportunity of thanking the numerous members of the nursing world—many of them quite unknown to me—for their kind letters of sympathy and good wishes for the success of my new undertaking.

### A CONTRADICTION.

Miss M. W. Lea, matron of the High Wycombe and Earl of Beaconsfield Memorial Cottage Hospital, writes : I shall be glad if you will contradict the statement made in your last issue that Miss G. M. Lloyd has been appointed matron of the High Wycombe Hospital. I have been matron of this hospital for the last nine years, and have no intention at present of giving up the post. Miss Lloyd has, I believe, been appointed to the Isolation Hospital, High Wycombe.

### Appointments.

**[No charge is made for announcements under this head, and we are always glad to receive and publish appointments. The information, to insure accuracy, should be sent from the nurses themselves, and we cannot undertake to correct official announcements which may happen to be inaccurate. It is essential that in all cases the school of training should be given.]**

Bethnal Green Infirmary.—Miss Minnie Thompson has been appointed sister. She was trained at Southwark Infirmary, East Dulwich, where she has since been sister. She has also done private nursing.

Bradford Eye and Ear Hospital.—Miss Frances Harrison has been appointed sister. She was trained at the General Infirmary, Leeds, and has since been sister, and sister of the ophthalmic ward in the same institution. She was subsequently sister and night superintendent at the Victoria Hospital, Chelsea, London.

Burton-upon-Trent Workhouse Infirmary.—Miss Elizabeth Moss has been appointed superintendent nurse. She was trained at Birmingham Workhouse Infirmary, and she has since been sister of the maternity ward of Leeds Workhouse Infirmary.

City Hospital for Infectious Diseases, Newcastle-on-Tyne.—Miss Vere Adeline Pagan has been appointed day sister. She was trained at the Adelaide Hospital, Dublin, and has since been sister at the Enfield Hospital, and the Royal Hants Hospital, Southampton.

Colne and Holme Fever Hospital, Meltham.—Miss Mary E. Clark has been appointed charge nurse. She was trained at Belvidere Hospital, Glasgow, and she has since been charge nurse in the same institution.

Cottage Hospital, Fleetwood.—Miss K. Godson has been appointed matron. She was trained at the Queen's Hospital, Birmingham, has since been sister at the County Hospital, Newport, Mon., and sister in charge of the operating theatre and male wards at the Clayton Hospital, Wakefield.

Crumpsall Infirmary, Manchester.—Miss Frances Ann Mycock has been appointed night superintendent. She was trained at Crumpsall Infirmary, and has since been ward sister and theatre sister at the same institution.

Eastbourne Sanatorium.—Miss L. U. Cuidesey has been appointed charge nurse. She was trained at the Borough Hospital, Cambridge, and has since been staff nurse at the Sanitary Hospital, Bournemouth, and the Borough Hospital, Bolton.

Edinburgh Royal Infirmary.—Miss Jane Bell has been appointed assistant lady superintendent of nurses. She was trained at the Royal Prince Alfred Hospital, Sydney, New South Wales, and has since held several posts in the Australian colonies.

Grantham Hospital.—Miss Florence May Eaton has been appointed matron. She was trained at Birmingham General Hospital, and has since been sister of the male accident wards in the same institution for five years

Indian Nursing Association.—Miss M. K. Smith, Miss K. Thorburn, Miss Williams, and Miss Forward have been appointed nursing sisters. Miss Smith was trained at St. Bartholomew's Hospital and has since worked on the staff of the Registered Nurses' Society. She holds the Central Midwives Board certificate. Miss Thorburn was trained at the Derbyshire Royal Infirmary, and has since been sister at the Royal Victoria Hospital, Bournemouth, and holiday sister at the Hospital for Women, Soho Square,

London. She holds the Central Midwives Board certificate. Miss Williams was trained at the West Kent General Hospital, Maidstone, and holds the Central Midwives Board certificate. Miss Forward was trained at the London Hospital, and holds the Central Midwives Board certificate.

Jaffray Hospital, Birmingham.—Miss M. Williams has been appointed sister. She was trained at Kidderminster Infirmary and Children's Hospital. She has since been staff nurse at Birmingham General Hospital, sister at Kidderminster Infirmary, and sister at West Ham and East London Hospital.

Liverpool Hospital for Women.—Miss Marion Gratson has been appointed night sister. She was trained at Aylesbury Hospital and Guy's Hospital, London. She has since been staff nurse at Guy's Hospital, where she has done holiday sister's duty. She has also taken holiday duty at the East London Hospital for Women and Children.

Manchester Southern Hospital.—Miss Pauline Rochford has been appointed staff nurse. She was trained at the General Infirmary, Oldham, Lancashire, and has since been staff nurse and out-patient and casualty nurse in the same institution.

Salisbury Hostel and Maternity Hospital, Rhodesia. Miss E. M. Halliwell has been appointed Lady Superintendent. She was trained at the Royal Infirmary, Newcastle-on-Tyne, after which she served in South Africa as a sister of the Army Nursing Service Reserve. She has since been matron of the Samaritan Hospital for Women, Liverpool. She holds the certificates of the British Gynæcological Society, the Royal Incorporated Society of Trained Masseuses, and the Central Midwives Board.

Southwark Infirmary.—Miss M. A. Walmsley has been appointed night superintendent. She was trained at the London Hospital and has since been ward sister at the Bethnal Green Infirmary.

Sunderland Infirmary.—Miss Kathleen S. Stewart has been appointed night superintendent. She was trained at Sunderland Infirmary, and for midwifery at the Royal Maternity and Simpson Memorial Hospital, Edinburgh. She has since been staff nurse in charge of the midwifery and district nursing staff, nurse in charge of the wards and operating theatre in the Deaconess Hospital, Edinburgh, holiday sister and holiday assistant matron at the Royal Hospital for Sick Children, Edinburgh. She holds the certificates of the Central Midwives Board and the Scottish Board of Obstetric Nursing.

Woodford Jubilee Hospital, Essex.—Miss Kathleen Fitzgerald has been appointed staff nurse. She was trained at the London Hospital and the Royal Orthopædic Hospital, London.

### The Nurses' Bookshelf.

Questions and Answers on Nursing. By J. W. Martin, M.D. 5½ × 4. (Baillière, Tindall, and Cox, King William Street, Strand.)

We have perused this little book with pleasure. The questions and answers are given in such simple language that no difficulty can arise in understanding them. The trained nurse or pupil in an ambulance class equally will find much to instruct and distinct practical aid in nursing and the rendering of first-aid. The subjects treated include the preparation and furnishing in cases of infectious disease, warming and ventilation, infection and disinfection, points connected with operations, and the application of local remedies. We can cordially recommend the book.

### Christmas Presents.

### CADBURY'S CHOCOLATE.

With regard to chocolate, the name of Cadbury is a guarantee that the boxes bearing it contain none but the very best sweetmeats, and the designs for Christmas this year are simply unlimited. The guinea cabinets in their first stage are filled with delicious chocolates of every shape, size, and flavour, and afterwards they can be utilised for jewel-cases, as they are daintily made of plush and fitted with lock and key. For less than a quarter of this sum (5s.) there is an endless variety to choose from, and notable are the handkerchief- and glove-boxes in leather and other materials, filled just now with sweets, but admirably suited to more prosaic purposes later on, deserve a front place. Charming specimens can be had for half a crown each. Even 3d. is not too low a sum for Messrs. Cadbury to produce a pretty little box of quaint design with some chocolates inside. The chocolate biscuits are such favourites with all children that it is hardly needful to allude to them, but they are a specially healthy sweetmeat, and the most delicate digestion is not upset with these because the crisp biscuits takes away any suggestion of richness which the chocolate may possess.

### A NEW HOT-WATER BOTTLE.

No house, whether its inmates are well or ill, should ever be without a hot-water bottle. It is not only a great source of comfort, but also by the steady application of heat it is frequently the means of staving off threatened illness and of materially diminishing pain. But an unreliable bottle is almost worse than none at all, and it is a pleasure to be able to call attention to the "Celerio" hot-water bottle manufactured by Messrs. J. B. Brooks and Company, Limited, Great Charles Street, Birmingham. There is no losing the screw stopper, as it does not possess one, and the patent hot-water valve makes the bottle absolutely unleakable. Moreover, it is so constructed that no bubbling over can take place when it is being filled, which in itself is a great advantage, as the hands need never be scalded. Patients will appreciate the fact that when the bottle is in use the valve and neck are not felt, so that it can be lain upon with perfect ease. The price is not higher than that charged for the ordinary make of hot-water bottle, and there is only one quality—the best.

### CHILDREN'S TOYS.

Many kind hearts, anxious to cheer the little ones in hospitals and elsewhere at Christmas time, are debarred from sending toys because they feel that the trouble of selecting and packing is more than they have time to undertake. In such circumstances Messrs. Toler Brothers, Limited, have come to the rescue. For twelve stamps forwarded to them at Savoy Corner, Thames Embankment, Strand, W.C., they will send a big box of ten toys, carriage paid, to any address desired—and, moreover, they are capital value for the money.

### TRAVEL NOTES AND QUERIES.

By our Travel Correspondent.

Tour to Italian Cities.—Under the auspices of Miss L. M. Davidson private social tours for gentlewomen to Italian cities have been arranged to take place in January. The date of starting has not yet been fixed, but it will not be before the 15th This will be Miss Davidson's 18th expedition, and, as on former occasions, the arrangements are of a very complete character, ample time being allowed to visit all the most famous cities in Italy, whether the tour be for the minimum period of twenty-five days or the maximum period of forty days. The inclusive terms are moderate, and full particulars will be given on application to Miss Davidson, who, as usual, accompanies and acts as cicerone to the party, at her address, 29 Dafforne Road, Upper Tooting, S.W.

### Notes and Queries.

**REGULATIONS.**

**The Editor is always willing to answer in this column, without any fee, all reasonable questions, as soon as possible.**

**But the following rules must be carefully observed.**

**1. Every communication must be accompanied by the name and address of the writer.**

**2. The question must always bear upon nursing, directly or indirectly.**

**If an answer is required by letter a fee of half-a-crown must be enclosed with the note containing the inquiry.**

*Delicate Girl wants Post.*

(131) Can you recommend a place in country institution for a young lady, 22, musical and refined, and rather delicate, who could undertake light household duties, secretarial, etc.?—*Nurse C.*

We think that the only course is to advertise.

*Probationer of 21 Years.*

(132) Can you please tell me where, at the age of 21 years, I could receive training as a probationer with salary?—*Richmond.*

None but children's hospitals and some of the poor-law institutions receive probationers so young. You had better procure "How to Become a Nurse," 2s. 4d. post free, from the Scientific Press, 28 Southampton Street, Strand.

*Nursing in Cape Town.*

(133) How can I obtain an appointment as sister or staff nurse in or near Cape Town?—*Stratford.*

Write to Somerset Hospital, Cape Town, S.A. There is no South African nursing journal.

*Vaccination.*

(134) Would vaccination performed on a lying-in woman on the fourth day have any ill effects?—*Anxious.*

This is a question for a medical man.

*Coaching in Midwifery.*

(135) I have a Queen Charlotte's certificate for midwifery. I wish to pass the C.M.B. examination. Where could I be coached, and where can I get practical teaching in return for services, etc.?—*Mollie.*

Write for advice to The Midwives Institute, 12 Buckingham Street, Strand, W.C.

*Nursing Abroad.*

(136) Is there an agency for nursing abroad?—*Clapham.*

No; but there are several nursing institutions abroad, of which you will find addresses in "How to Become a Nurse," The Scientific Press, 28 Southampton Street, Strand, 2s. 4d. post free. We fear you will be too late to secure a post this season.

*A Colonial Nurse.*

(137) Can you tell me where I could be treated in London for kidney trouble?—*Inquirer.*

We feel sure if you write to the Secretary of Guy's Hospital, London Bridge, E.C., or other hospitals, you will be received if you explain your case.

*Rescue Work.*

(138) Is it necessary to have any training for rescue work?—*Hatfield.*

Certainly you should have training. Write to the Church Army, Edgware Road, W., where you will receive excellent advice, and probably assistance.

*Nursing in Paris.*

(139) Can you tell me of any private nursing association in Paris where one can be taken on the co-operative system?—*Dainé.*

We know of no such institution. But in "Medical Homes for Private Patients," price 6d. post free, to be obtained from The Scientific Press, 28 Southampton Street, Strand, W.C., you will find a list of English doctors in Paris, and they may be able to tell you if there is an opening for an English nurse.

**Handbooks for Nurses.**

| | Post Free. |
|---|---|
| "A Handbook for Nurses." (Dr. J. K. Watson) ... | 5s. 4d. |
| "Nurses' Pronouncing Dictionary of Medical Terms" | 2s. 0d. |
| "Art of Massage." (Creighton Hale) ... ... ... | 6s. 0d. |
| "Surgical Bandaging and Dressings." (Johnson Smith.) ... ... ... ... ... ... ... | 2s. 0d. |
| "Hints on Tropical Fevers." (Sister Pollard.) ... | 1s. 8d. |

Of all booksellers or of The Scientific Press, Limited, 28 & 29 Southampton Street, Strand, London, W.C.

### For Reading to the Sick.

HOPE'S STRENGTH.

Be strong to hope, O heart!
Though day is bright,
The stars can only shine
In the dark night.
Be strong, O heart!
Look to the light.
Be strong to bear, O heart!
Nothing is vain,
Strive not, though life is care,
And God sends pain.
Heaven is above, and there
Rest will remain.

*A. A. Proctor.*

If our faith be true, we shall be content to know His way, and how to gain grace to keep it. We will mark the steps of Christ, sure that though the way be hard now, it leads to where He is. We will use the helps of which God tells, sure that though we cannot trace God's work, He keeps His word to those who obey Him. We will go on to the end, walking by faith, and seeking things not seen as yet. If we keep God's way, He will keep our souls, till by the way of well-doing we reach the land of promise, of which He has made us heirs in Christ. God will lead us, if we will be led, so that we shall know where light and safety are to be found. He will give faith to trust His leading. He will bring us out of the mists of our low state into the pure light of His truth. He will hold our hand, as we shrink in fear and doubt, cheering our souls with words of kindness. He will teach us how to tread in the rough ways, how to be safe from falling as we mount steadily. He will point us upwards, and warn us when we would look back. He will uphold us when we tire. The truth of Christ shall be our light, and the grace of Christ shall be our strength. The Rock shall seem higher each day; but we shall be higher too. So shall it be for ever. We ask for grace to shine on us, that the light of God's countenance may help us to lift up our heads, and that Divine love may lead us ever nearer. We plead with lowly trust the promises given to us in Christ. We join with Him Who pleads on high. We seek that He may dwell in us; and to that end we receive with thankful gladness the sacramental gifts by which our oneness with Him is made sure. We love the means and places wherein He teaches us to find his special presence, and know how true He is to the word in which He bids us trust. So, step by step, He leads us on in paths of righteousness for His Name's sake; and we press bravely on, through any darkness, towards the holy hill, where nothing clouds the shining of God's love.—*Anon.*

Grant us Thy peace when shadows fall,
When darkness deepens over all;
In fearless faith to sleep awhile—
In dreams behold Thy loving smile.
Grant us Thy life, O Living Lord—
The Life in God by Thee restored;
To live and move alone in Thee—
By Thy great love for ever free.

*Kingsland.*

